# Herpes simplex virus type 1 and type 2 in the Netherlands: seroprevalence, risk factors and changes during a 12-year period

**DOI:** 10.1186/s12879-016-1707-8

**Published:** 2016-08-02

**Authors:** Petra J. Woestenberg, Jeroen H. T. Tjhie, Hester E. de Melker, Fiona R. M. van der Klis, Jan E. A. M van Bergen, Marianne A. B. van der Sande, Birgit H. B. van Benthem

**Affiliations:** 1 Center for Infectious Disease Control, National Institute for Public Health and the Environment (RIVM), Bilthoven, The Netherlands; 2 Department of Medical Microbiology, Laboratory for Pathology and Medical Microbiology (PAMM), Veldhoven, The Netherlands; 3 STI AIDS Netherlands, Amsterdam, The Netherlands; 4 Department of General Practice, Academic Medical Center, Amsterdam, The Netherlands; 5 Julius Center, University Medical Center Utrecht, Utrecht, The Netherlands

**Keywords:** HSV-1, HSV-2, Herpes simplex virus, Genital herpes, Seroprevalence, Public health

## Abstract

**Background:**

Genital herpes results in considerable morbidity, including risk of neonatal herpes, and is increasingly being caused by Herpes Simplex Virus (HSV) type 1. Possibly children are less often HSV-1 infected, leaving them susceptible until sexual debut. We assessed changes in the Dutch HSV-1 and HSV-2 seroprevalence over time and determinants associated with HSV seropositivity.

**Methods:**

We used data from two population-based seroepidemiological studies conducted in 1995–6 and 2006–7 with a similar study design. Serum samples of 6 months to 44-year-old participants were tested for type-specific HSV antibodies using HerpesSelect® with a cut-off level of >1.10 for seropositivity. Age and sex-specific HSV-1 and HSV-2 seroprevalence was weighted for the Dutch population. Logistic regression was performed to investigate determinants associated with HSV seropositivity.

**Results:**

Overall, weighted HSV-1 seroprevalence was significantly lower in 2006–7 [42.7 % 95 % confidence interval (CI) 39.9-45.4] than in 1995–6 (47.7 % 95 % CI 44.8-50.7), especially among 10- to 14-year-olds. Overall, weighted HSV-2 seroprevalence remained stable: 6.8 % in 1995–6 and 6.0 % in 2006–7. Adults who ever had sexual intercourse were more often seropositive for HSV-1 [adjusted Odds Ratio (aOR) 1.69 95 % CI 1.33-2.16] and HSV-2 (aOR 2.35 95 % CI 1.23-4.52). Age at sexual debut was the only sexual risk determinant associated with HSV-1 seropositivity.

**Conclusions:**

Because of the lower HSV-1 seroprevalence in 2006–7 compared to 1995–6, more adults are susceptible to genital HSV-1, including women of reproductive age. Given the higher risk of neonatal herpes when HSV is acquired during pregnancy, prevention and control measures during pregnancy also targeting HSV-1, are important.

**Electronic supplementary material:**

The online version of this article (doi:10.1186/s12879-016-1707-8) contains supplementary material, which is available to authorized users.

## Background

Herpes Simplex Virus (HSV) can cause orolabial and genital infections. Most HSV infections are asymptomatic, but when symptoms occur, they consist primarily of ulcerative lesions at the site of infection. HSV infections remain lifelong and are characterized by latency and intermitted (sub)clinical reactivity and viral shedding [1]. HSV is highly prevalent with a worldwide estimate of approximately 3.6 billion people till the age of 49 infected orolabially and half a billion infected genitally [2].

Controlling HSV infections is of public health relevance. When transmitted vertically, HSV can cause neonatal herpes. Although occurring rarely (4.7/100,000 livebirths in the Netherlands [3]), neonatal herpes can have serious consequences such as neurological damage and death. Other complications of HSV infections include encephalitis and aseptic meningitis [1]. Moreover, genital HSV infections are associated with an increased risk of acquiring and transmitting human immunodeficiency virus (HIV) [4, 5].

There are two types of HSV: HSV-1 and HSV-2. HSV-1 has traditionally been characterized by childhood transmission causing orolabial lesions and HSV-2 by sexual transmission causing genital herpes. However, the epidemiology of HSV is changing and genital herpes is increasingly being caused by HSV-1 in industrialized countries [6–9]. This leads to HSV-1 being the main cause of primary genital herpes in some countries [10–12] and to an important role of HSV-1 in neonatal herpes [3].

A possible explanation for the increasing contribution of HSV-1 in genital herpes is that children are less often exposed to HSV-1, leaving them susceptible to genital HSV-1 [13]. Indeed, different studies observed a decline in HSV-1 seropositivity among children over time [14, 15].

For prevention and control strategies, it is important to monitor the epidemiology of HSV infections. In the Netherlands, a good picture of HSV-1 and HSV-2 in the general population and changes over time is lacking. By comparing two large-scale population-based seroepidemiological studies conducted in 1995–6 and 2006–7, we investigated changes in age and sex-specific HSV-1 and HSV-2 seroprevalence in the general Dutch population. We also investigated determinants associated with HSV-1 and HSV-2 seropositivity, including sexual risk behavior.

## Methods

### Study design and population

We used data from two cross-sectional population-based seroepidemiological studies, conducted in the Netherlands in 1995–6 (Pienter-1) and 2006–7 (Pienter-2). The study designs were similar and described in detail elsewhere [16, 17]. Briefly, 40 municipalities equally distributed over 5 geographical regions were randomly selected proportional to their population size. An age-stratified sample was drawn from the population register and invited to donate blood and fill in a questionnaire at home. In Pienter-2, migrants (people with at least one parent born abroad) were oversampled. The participation rates for Pienter-1 and Pienter-2 were 55 % and 32 %, respectively. Pienter-1 was approved by the Medical Ethical Committee of Netherlands Organization for Applied Scientific Research (TNO) in Leiden. Pienter 2 was approved by the Medical Ethics Testing Committee of the foundation of therapeutic evaluation of medicines (METC-STEG) in Almere (clinical trial number: ISRCTN 20164309). All participants gave written informed consent.

We used data from the nationwide sample from all 6 months to 44-year-old participants. Children younger than 6 months were excluded because of the possible presence of maternally derived antibodies.

### Laboratory methods

Serum samples were stored at −80 °C until analyses (in 2000–1 for Pienter-1 and 2013 for Pienter-2 samples). Type-specific antibodies were determined with a commercial indirect HSV-1 (gG1) and HSV-2 (gG2) antibody assay (HerpesSelect®, Focus Technologies, Dypress, CA, USA) according to the manufacturer instructions [18, 19]. Cut-off levels were >1.10 for seropositivity and <0.90 for seronegativity. Equivocal samples (1.8 % of all samples for HSV-1 and 0.6 % for HSV-2) were classified as negative for all analyses [15, 20].

### Statistical analyses

All statistical analyses were corrected for the complex survey design, by using the Taylor series variance estimation [21]. We calculated the overall and age- and sex-specific HSV-1 and HSV-2 seroprevalence and their joint distribution. By assigning sampling weights to each sample, we calculated the seroprevalence of the general Dutch population in Pienter-1 and Pienter-2 to study changes over time. Weights were determined from the Dutch reference population of 1996 and 2007, taking into account gender, age, ethnicity and degree of urbanization of the participants’ residence. Differences in the weighted seroprevalence between Pienter-1 and Pienter-2 were calculated using Chi-square tests.

We performed logistic regression analyses to investigate determinants associated with HSV seropositivity. Since the rare event assumption was not met, Odds Ratios (OR) cannot be interpreted as relative risks [22]. The logistic regression analyses were unweighted; instead, all logistic regression analyses were adjusted for the variables that were considered in the weights (gender, age, ethnicity and degree of urbanization) to correct for oversampling and nonresponse [23].

Taking into account different risk behavior and possible different routes of exposure, analyses were performed separately for children (aged 6 months to 11 years), all adults (aged 17 to 44 years for Pienter-1 and 15 to 44 years for Pienter-2, because the questionnaire was distributed to participants aged 17 or older in Pienter-1 and to participants aged 15 or older in Pienter-2) and adults who ever had sexual intercourse. Because there were hardly any differences between Pienter-1 and Pienter-2 in determinants associated with seropositivity (Additional file 1), the studies were analyzed together.

For HSV-1, we considered as possible determinants: demographics [gender, age, ethnicity, generation of migrants, degree of urbanization and education level (for children, the education level of the parents was used)], social contact variables [number of household members, child in household attending day care and attending day care (for children only)] and whether someone ever had sexual intercourse (for adults only). Among adults who ever had sexual intercourse, we investigated sexual risk behavior: number of recent partners (in the past year for Pienter-1 and 6 months for Pienter-2), sexual preference based on sexual behavior, self-reported history of specific sexually transmitted infections (STI), age of sexual debut and condom use with steady and casual partners in the past 6 months (Pienter-2 only).

For HSV-2, analyses were performed for adults only, since children were rarely seropositive. Variables included were demographics, whether someone ever had sexual intercourse and sexual risk behavior (for adults who ever had sexual intercourse only).

We performed logistic regression analyses separately for all variables described above adjusted for gender, age, ethnicity and degree of urbanization. All variables were included in further multivariable analyses.

Among adults who ever had sexual intercourse, we calculated the proportion reporting a history of genital herpes by HSV serostatus, stratified by Pienter study. These percentages were unweighted in absence of a reference population by HSV serostatus. Analyses were performed using SAS 9.3 with a significance level of *p* < 0.05.

### Sensitivity analyses

In sensitivity analyses, we repeated the analyses with equivocal samples classified as positive. In addition, we performed a sensitivity analysis for the relation between sexual risk determinants on HSV-1 seropositivity restricted to Native Dutch adults (i.e., excluding migrants), to increase the proportion of genital HSV-1 infections. The HSV-1 seroprevalence among migrants in the Netherlands is very high at a young age [24] probably related to orolabial infections. Last, we repeated the analyses for number of sex partners where we converted the number of partners in the past year (Pienter-1 variable) to the number of partners in the past 6 months to match the variable of Pienter-2. We did this by rounding up the number of partners in the past year divided by 2.

## Results

### Study population

Sufficient serum was left for 4180 of the 4943 participants aged 6 months to 44 years in Pienter-1 and for 3757 of the 3928 participants in Pienter-2. Of the 7937 participants included overall, 2887 (36.4 %) were children aged 6 months to 11 years and 4276 (53.9 %) were adults aged 15/17 to 44 years. Of all adults, 3600 (84.2 %) ever had sexual intercourse.

Due to oversampling of migrants in Pienter-2, participants were less often native Dutch and more often living in a highly urbanized area in Pienter-2 compared to Pienter-1. Children in Pienter-2 were less often living with five or more people, more often living with a child attending day care and more often attending day care themselves. Adults in Pienter-2 were more often highly educated, more often living with a child attending day care and less often sexually experienced (Table 1).

**Table 1:** Characteristics of the study population (children and adults^a^) of Pienter-1 (1995–6) and Pienter-2 (2006–7)

	Children^a^				Adults^a^			
	Pienter-1		Pienter-2		Pienter-1		Pienter-2	
	N	%	N	%	N	%	N	%
Total	1403		1484		2253		2023	
Gender								
Men	755	53.81	754	50.81	1006	44.65	815	40.29
Women	648	46.19	730	49.19	1247	55.35	1208	59.71
Age (median)	5		5		32		30	
Ethnicity^b^								
Native Dutch	1201	85.60	897	60.44	2019	89.61	1689	83.49
Western, other	74	5.27	57	3.84	131	5.81	152	7.51
Moroccan/Turkish	57	4.06	202	13.61	47	2.09	50	2.47
Surinamese/Aruban/Antillean	24	1.71	164	11.05	27	1.20	50	2.47
Non-Western, other	47	3.35	164	11.05	29	1.29	82	4.05
Generation of migrant^b^								
Native Dutch	1201	85.60	897	60.44	2019	89.61	1689	83.49
1st generation	41	2.92	286	19.27	123	5.46	174	8.60
2nd generation	161	11.48	301	20.28	111	4.93	160	7.91
Degree of urbanization								
Very high	160	11.40	416	28.03	233	10.34	379	18.73
Less high	1243	88.60	1068	71.97	2020	89.66	1644	81.27
Education level^c^								
Moderate or low	955	68.07	1014	68.33	1794	79.63	1396	69.01
High	421	30.01	431	29.04	436	19.35	601	29.71
Unknown	27	1.92	39	2.63	23	1.02	26	1.29
Household								
1-2 persons	19	1.35	46	3.10	724	32.13	643	31.78
3-4 persons	852	60.73	888	59.84	1086	48.20	1000	49.43
> = 5 persons	520	37.06	515	34.70	408	18.11	356	17.60
Unknown	12	0.86	35	2.36	35	1.55	24	1.19
Child in household attending day care								
No	1193	85.03	1154	77.76	1972	87.53	1694	83.74
Yes	184	13.11	278	18.73	237	10.52	295	14.58
Unknown	26	1.85	52	3.50	44	1.95	34	1.68
Child attending day care								
No	1091	77.76	1058	71.29	-	-	-	-
Yes	281	20.03	382	25.74	-	-	-	-
Unknown	31	2.21	44	2.96	-	-	-	-
Ever had sexual intercourse								
No	-	-	-	-	206	9.14	266	13.15
Yes	-	-	-	-	1932	85.75	1668	82.45
Unknown	-	-	-	-	115	5.10	89	4.40

Numbers and percentages were unweighted

^a^Children were aged 6 months to 11 years and adults were aged 17 to 44 years in Pienter-1 and 15 to 44 years in Pienter-2

^b^In Pienter-2, migrant populations (people with at least one parent born abroad) were oversampled

^c^For children, the education level of the parents was used

### Weighted seroprevalence

**Fig. 1:**
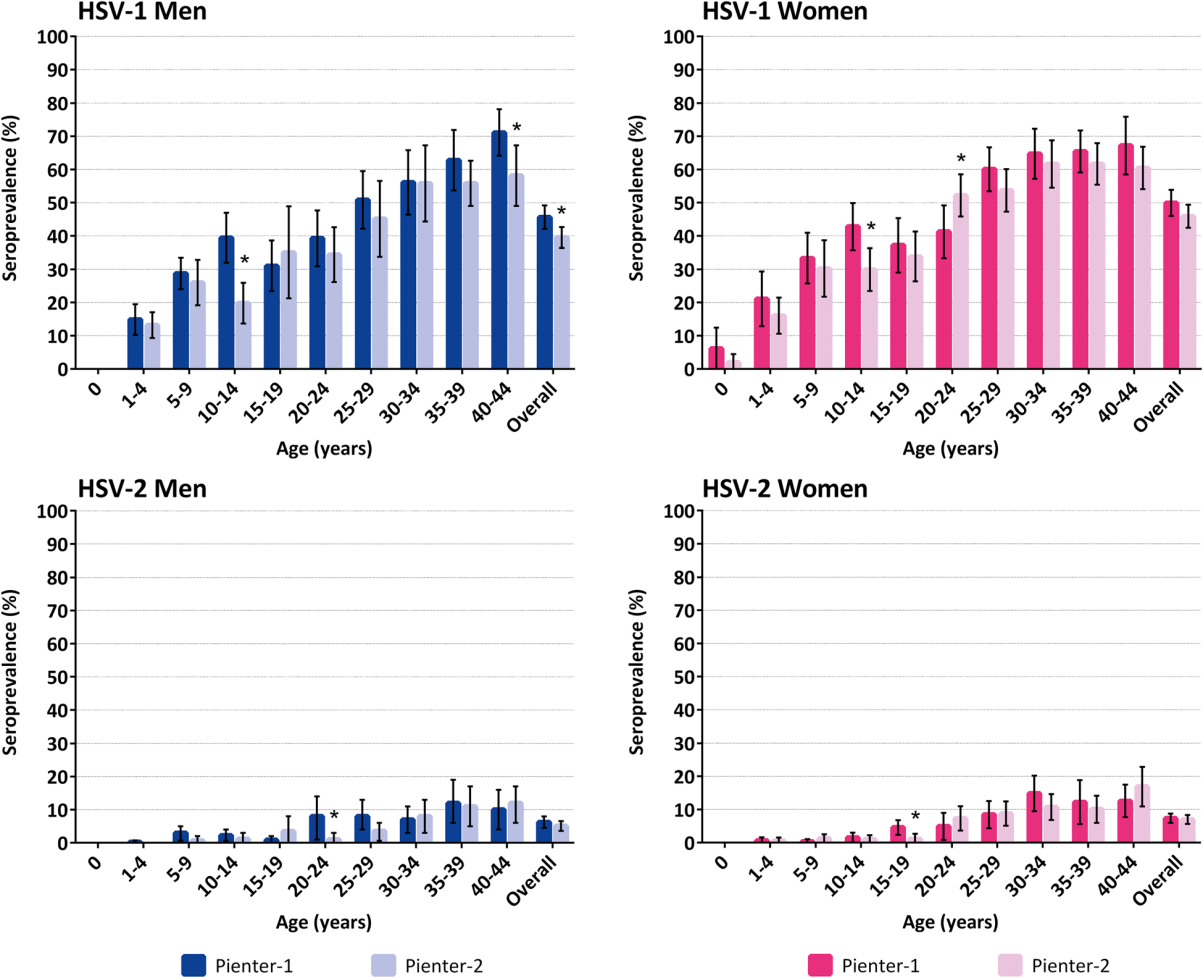
Weighted seroprevalence of HSV-1 and HSV-2 by gender, age and Pienter study. * Difference in seroprevalence between Pienter-1 and Pienter-2 is statistically significant (*p* < 0.05)

The overall HSV-1 seroprevalence of the general Dutch population in Pienter-2 was 42.7 % [95 % confidence interval (CI) 39.9-45.4] and was lower than the overall seroprevalence of the general population in Pienter-1 (47.7 %, 95 % CI 44.8-50.7, *p* = 0.01). The weighted HSV-1 seroprevalence in Pienter-2 was, compared to Pienter-1, significantly lower among 10- to 14-year-olds and significantly higher among 20- to 24-year-old women (Fig. 1).

The overall HSV-2 seroprevalence of the general Dutch population in Pienter-2 was 6.0 % (95 % CI 4.8-7.2) and comparable to the overall seroprevalence of the general population in Pienter-1 (6.8 %, 95 % CI 5.6-8.0, *p* = 0.4). There were some age and sex-specific changes. Among 20- to 24-year-old men and 15- to 19-year-old women, the weighted HSV-2 seroprevalence was lower in Pienter-2 compared to Pienter-1 (Fig. 1). The joint distribution of HSV-1 and HSV-2 seroprevalence in the general population is presented in Additional file 2.

### Determinants associated with seropositivity

Adjusted for all variables, children in Pienter-2 were less often HSV-1 seropositive than children in Pienter-1 (adjusted OR (aOR) 0.68 95 % CI 0.54-0.85) (Table 2). Older children, females, children with a non-Western ethnicity and children with moderately or low educated parents were more often HSV-1 seropositive. Children attending day care themselves were also more often HSV-1 seropositive, while living with children attending day care was not associated with HSV-1 seropositivity.

**Table 2:** Logistic regression analyses to investigate demographic and social determinants associated with HSV-1 and HSV-2 seropositivity

	HSV-1				HSV-2	
	Children^a^		Adults^a^		Adults^a^	
	OR [95 % CI]^b^	aOR [95 % CI]	OR [95 % CI]^b^	aOR [95 % CI]	OR [95 % CI]^b^	aOR [95 % CI]
Pienter						
Pienter-1	Ref.	Ref.	Ref.	Ref.	Ref.	Ref.
Pienter-2	**0.69 [0.55-0.86]**	**0.68 [0.54-0.85]**	**0.79 [0.66-0.94]**	**0.80 [0.67-0.96]**	0.94 [0.71-1.25]	0.90 [0.68-1.21]
Gender						
Men	Ref.	Ref.	Ref.	Ref.	Ref.	Ref.
Women	**1.28 [1.09-1.51]**	**1.32 [1.12-1.56]**	**1.22 [1.07-1.40]**	**1.20 [1.04-1.37]**	**1.46 [1.22-1.74]**	**1.46 [1.22-1.75]**
Age (continuously)	**1.21 [1.18-1.23]**	**1.23 [1.20-1.26]**	**1.06 [1.05-1.07]**	**1.05 [1.04-1.06]**	**1.05 [1.04-1.07]**	**1.04 [1.03-1.06]**
Ethnicity						
Native Dutch	Ref.	Ref.	Ref.	Ref.	Ref.	Ref.
Western, other	1.01 [0.64-1.59]	1.03 [0.66-1.62]	**1.35 [1.06-1.70]**	**1.42 [1.12-1.80]**	1.23 [0.85-1.78]	1.21 [0.84-1.76]
Moroccan/Turkish	**4.02 [2.80-5.78]**	**4.00 [2.74-5.83]**	**15.80 [8.15-30.64]**	**16.30 [8.15-32.61]**	0.78 [0.35-1.72]	0.84 [0.37-1.92]
Surinamese/Aruban/Antillean	**2.24 [1.67-3.01]**	**2.55 [1.89-3.43]**	**2.46 [1.52-4.00]**	**2.74 [1.69-4.46]**	**1.85 [1.01-3.37]**	**2.02 [1.11-3.66]**
Non-Western, other	**2.45 [1.75-3.42]**	**2.60 [1.78-3.78]**	**4.47 [2.65-7.56]**	**4.79 [2.74-8.37]**	**1.87 [1.05-3.30]**	**1.98 [1.12-3.49]**
Generation of migrant^c^						
Native Dutch	Ref.	-	Ref.	-	Ref.	-
1st generation	**3.29 [2.36-4.57]**	-	**4.84 [3.49-6.70]**	-	**1.59 [1.11-2.28]**	-
2nd generation	**1.94 [1.48-2.53]**	-	**1.38 [1.04-1.83]**	-	1.05 [0.68-1.62]	-
Degree of urbanization						
Very high	1.12 [0.85-1.48]	1.16 [0.89-1.50]	0.90 [0.74-1.08]	0.97 [0.81-1.15]	1.07 [0.75-1.52]	1.04 [0.73-1.48]
Less high	Ref.	Ref.	Ref.	Ref.	Ref.	Ref.
Education level^d^						
Moderate or low	**1.43 [1.16-1.77]**	**1.44 [1.16-1.80]**	**1.26 [1.07-1.47]**	**1.23 [1.05-1.45]**	**0.69 [0.55-0.88]**	**0.70 [0.55-0.89]**
High	Ref.	Ref.	Ref.	Ref.	Ref.	Ref.
Unknown	**2.06 [1.19-3.57]**	1.53 [0.79-2.98]	1.35 [0.67-2.70]	1.80 [0.85-3.82]	1.10 [0.43-2.85]	1.14 [0.41-3.12]
Household						
1-2 persons	Ref.	Ref.	Ref.	Ref.	-	-
3-4 persons	1.58 [0.84-2.95]	1.57 [0.84-2.93]	**1.17 [1.02-1.34]**	1.14 [0.99-1.31]	-	-
> = 5 persons	1.64 [0.86-3.13]	1.69 [0.89-3.20]	1.08 [0.89-1.30]	1.10 [0.90-1.34]	-	-
Unknown	**3.94 [2.17-7.16]**	**3.94 [1.93-8.04]**	1.15 [0.65-2.03]	**2.04 [1.03-4.04]**	-	-
Child in household attending day care						
No	Ref.	Ref.	Ref.	Ref.	-	-
Yes	0.95 [0.71-1.28]	0.95 [0.71-1.26]	1.17 [0.98-1.41]	1.14 [0.94-1.39]	-	-
Unknown	1.41 [0.95-2.08]	1.20 [0.58-2.50]	**0.48 [0.29-0.78]**	**0.33 [0.18-0.58]**	-	-
Child attending day care						
No	Ref.	Ref.	-	-	-	-
Yes	**1.41 [1.07-1.85]**	**1.50 [1.12-2.01]**	-	-	-	-
Unknown	1.31 [0.87-1.97]	0.79 [0.36-1.73]	-	-	-	-
Ever had sexual intercourse						
No	-	-	Ref.	Ref.	Ref.	Ref.
Yes	-	-	**1.63 [1.29-2.06]**	**1.69 [1.33-2.16]**	**2.47 [1.28-4.74]**	**2.35 [1.23-4.52]**
Unknown	-	-	1.38 [0.98-1.95]	1.39 [0.97-1.99]	**2.43 [1.13-5.22]**	**2.22 [1.03-4.81]**

Logistic regression analyses were unweighted, corrected for the complex survey design

In bold: OR is statistically significant (*p* < 0.05)

*HSV* herpes simplex virus; *OR* odds ratio; *aOR* adjusted odds ratio; *CI* confidence interval; *Ref* reference

^a^Children were aged 6 months to 11 years and adults were aged 17 – 44 years in Pienter-1 and 15 – 44 years in Pienter-2

^b^OR adjusted for: gender, age, ethnicity and degree of urbanization

^c^Not adjusted for ethnicity and not included in multivariable analyses

^d^For children, the education level of the parents was used

Determinants associated with HSV-1 seropositivity among adults were comparable to those among children: being female, being older, having a non-Dutch ethnicity and being moderately or low educated (Table 2). Adults who ever had sexual intercourse were more often HSV-1 seropositive than adults who never had sexual intercourse (aOR 1.69 95 % CI 1.33-2.16).

Participants who were older at sexual debut were less often HSV-1 seropositive (Table 3). Other sexual risk determinants were not associated with HSV-1 in the multivariable analyses. Participants with a self-reported history of genital herpes had a higher odds of being HSV-1 seropositive compared to participants who reported no history of STI (aOR 1.84), however this was not statistically significant (95 % CI 0.75-4.53).

**Table 3:** Logistic regression analyses to investigate sexual risk determinants associated with HSV-1 and HSV-2 seropositivity^a^

		HSV-1		HSV-2	
	N (%)	OR [95 % CI]^b^	aOR [95 % CI]^c^	OR [95 % CI]^b^	aOR [95 % CI]^c^
Number of recent partners^d^					
0 partners	226 (6.28)	Ref.	Ref.	Ref.	Ref.
1 partners	3031 (84.19)	**1.39 [1.05-1.83]**	1.12 [0.71-1.75]	1.28 [0.81-2.04]	0.52 [0.24-1.12]
> = 2 partners	195 (5.42)	1.15 [0.75-1.74]	0.85 [0.50-1.46]	1.51 [0.82-2.78]	0.48 [0.21-1.12]
Unknown	148 (4.11)	**1.68 [1.10-2.59]**	1.34 [0.84-2.13]	1.27 [0.65-2.48]	0.78 [0.37-1.64]
Sexual preference					
Heterosexual	3242 (90.06)	Ref.	Ref.	Ref.	Ref.
Homo-/bisexual	47 (1.31)	1.10 [0.66-1.84]	1.23 [0.72-2.11]	**2.31 [1.23-4.35]**	1.94 [0.98-3.83]
Unknown	311 (8.64)	**0.78 [0.62-0.98]**	0.87 [0.60-1.26]	**0.58 [0.38-0.91]**	**0.35 [0.17-0.72]**
Self-reported history of STI					
No	3326 (92.39)	Ref.	Ref.	Ref.	Ref.
Yes, excluding genital herpes	123 (3.42)	1.18 [0.79-1.76]	1.18 [0.78-1.76]	**1.75 [1.06-2.90]**	1.66 [0.98-2.81]
Yes, genital herpes	29 (0.81)	1.88 [0.78-4.50]	1.84 [0.75-4.53]	**7.53 [3.20-17.69]**	**7.71 [3.19-18.60]**
Unknown	122 (3.39)	1.11 [0.73-1.68]	1.04 [0.69-1.58]	1.12 [0.60-2.09]	1.21 [0.66-2.22]
Age at sexual debut					
<=16 years	871 (24.19)	Ref.	Ref.	Ref.	Ref.
17-20 years	1703 (47.31)	**0.75 [0.63-0.89]**	**0.74 [0.62-0.88]**	1.20 [0.88-1.63]	1.22 [0.88-1.69]
> = 21 years	562 (15.61)	**0.62 [0.51-0.76]**	**0.64 [0.52-0.78]**	1.02 [0.74-1.41]	1.05 [0.74-1.49]
Unknown	464 (12.89)	0.89 [0.72-1.09]	0.85 [0.70-1.04]	0.91 [0.61-1.38]	0.96 [0.63-1.46]
Condom use steady partner^e^					
Consistent	219 (13.13)	Ref.	-	Ref.	-
Inconsistent	1068 (64.03)	0.96 [0.70-1.32]	-	1.12 [0.65-1.92]	-
Unknown/no steady partner	381 (22.84)	0.91 [0.63-1.30]	-	0.79 [0.43-1.45]	-
Condom use casual partner^e^					
Consistent	80 (4.80)	Ref.	-	Ref.	-
Inconsistent	68 (4.08)	1.25 [0.67-2.32]	-	0.30 [0.07-1.25]	-
Unknown/no casual partner	1520 (91.13)	1.04 [0.66-1.63]	-	0.62 [0.31-1.24]	-

Numbers and percentages were unweighted. Logistic regression analyses were unweighted, corrected for the complex survey design

In bold: OR is statistically significant (*p* < 0.05)

*HSV* herpes simplex virus; *OR* odds ratio; *aOR* adjusted odds ratio; *CI* confidence interval; *Ref* reference; *STI* sexually transmitted infection

^a^Only adults who ever had sexual intercourse were included. Adults were aged 17 to 44 years in Pienter-1 and 15 to 44 years in Pienter-2

^b^OR adjusted for: gender, age, ethnicity and degree of urbanization

^c^Adjusted for all variables including those presented in Table 2

^d^Number of partners in the past year for Pienter-1 and in the past 6 months for Pienter-2

^e^Condom use in the past 6 months. Available for Pienter-2 only

Determinants significantly associated with HSV-2 seropositivity among adults were: a female gender; an older age; a Surinamese, Aruban, Antillean or other non-Western ethnicity; and a high education level (Table 2). Sexually experienced adults were more often HSV-2 seropositive than adults who never had sexual intercourse (aOR 2.35 95 % CI 1.23-4.52).

Sexually experienced adults who were homo- or bisexual were more likely to be HSV-2 seropositive compared to heterosexual adults, however this was not statistically significant in the multivariable analyses (Table 3). Adults reporting a history of genital herpes were more often HSV-2 seropositive than adults reporting no history of STI (aOR 7.71 95 % CI 3.19-18.60). Condom use in the past 6 months was not significantly associated with HSV-2. The results of all sensitivity analyses were comparable to the original analyses (Additional files 3, 4, 5).

### Genital herpes

**Fig. 2:**
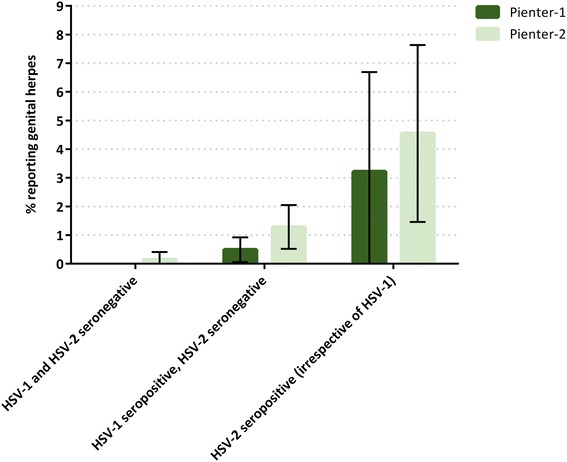
Proportion of adults reporting a history of genital herpes by HSV serostatus and Pienter study. Only adults who ever had sexual intercourse were included. Proportions are unweighted

Among all HSV-1 seropositive and HSV-2 seronegative adults who ever had sexual intercourse, 0.8 % reported a history of genital herpes. This increased from 0.5 % in Pienter-1 to 1.3 % in Pienter-2 (*p* = 0.06) (Fig. 2). Participants who were HSV-2 seropositive (irrespective of HSV-1 serostatus) more often reported a history of genital herpes (3.8 %).

## Discussion

In two population-based studies performed 12 years apart i.e., 1995–6 and 2006–7, we found that the overall HSV-1 seroprevalence decreased, while the HSV-2 seroprevalence remained stable. Especially among 10- to 14-year-olds, the HSV-1 seroprevalence was significantly lower in 2006–7 than in 1995–6. Adults who ever had sexual intercourse were more likely to be HSV seropositive, but age at sexual debut was the only sexual risk determinant associated with HSV-1 seropositivity.

There are some limitations. First, we were restricted to variables available in the Pienter studies. Variables related to HSV in other studies, like lifetime number of sex partners and oral sex, were not available and could therefore not be investigated [15, 25]. Second, the participation rate of 55 % in Pienter-1 and 32 % in Pienter-2 could have led to response bias. The participation rate differed by gender, age and ethnicity in both Pienter studies and by marital status and degree of urbanization in Pienter-1 [17, 26]. We tried to minimize possible bias by correcting for demographic variables by using weights or by including them as variables in the analyses. Third, as result of the cross-sectional design, we were unable to separate age and cohort effects. As HSV-1 and HSV-2 are lifelong infections, it is difficult to infer changes in seroincidence using seroprevalence in older age groups. Fourth, according to the manufacturer the specificity of the HerpesSelect® has increased with 1-2 % since 2000–1 when Pienter-1 samples were tested compared to 2013 when Pienter-2 samples were tested (personal communication), meaning less false positive samples for Pienter-2 compared to Pienter-1. This could have partially contributed to the differences between Pienter-1 and Pienter-2. Last, based on serology it is impossible to make a distinction between orolabial and genital infections if no symptoms are reported. We are therefore unable to draw conclusions about changes in genitally versus non-genitally acquired HSV-1 infections.

We observed an HSV-1 seroprevalence of 47.7 % in 1995–6 and 42.7 % in 2006–7 among 6 months to 44-year-olds, which is somewhat lower than the estimated HSV-1 seroprevalence for the European region (67-69 %) [2]. However, this European estimate was among 0- to 49-year-olds and not all countries were included, while there are large differences between counties, also within Europe. In a multi-country study, the age standardized HSV-1 seroprevalence of people aged 0 years and older ranged from 52 % in Finland to 84 % in Bulgaria [20].

The HSV-2 seroprevalence also differs widely between countries. The age standardized HSV-2 seroprevalence ranged from 4 % in England and Wales (>14 years old) to 24 % in Bulgaria (>11 years old) [20]. The HSV-2 seroprevalence in the total European region was estimated to be approximately 7 % among 15- to 49-year-olds [27]. In our study, the HSV-2 seroprevalence among 15- to 44-year-olds was 9.1 % in 1995–6 and 8.3 % in 2006–7, which is slightly higher than the European estimate.

As we hypothesized, we observed a lower HSV-1 seroprevalence among children in 2006–7 compared to 1995–6, possibly related to better hygiene. A decline in HSV-1 seroprevalence among young people was also observed in other countries. In England and Wales, the HSV-1 seroprevalence among 10- to 14-year-olds declined from 34 % in 1986–7 to 24 % in 1994–5 and in the US the HSV-1 seroprevalence among 14- to 19-year-olds declined from 43 % in 1976–80 to 30 % in 2005–10 [14, 15]. The decreased HSV-1 seroprevalence among children results in an increased age of infection and thus in higher susceptibility at sexual debut and might partly explain the increasing importance of HSV-1 in genital herpes described in the literature [6-9]. The rise in HSV-1 seroprevalence among 20- to 24-year-old women in our study could be explained by higher acquisition of genital HSV-1.

We found no trend in the HSV-2 seroprevalence. This is also found in the US were the HSV-2 seroprevalence among 14- to 49-year-olds was 17 % in 1999–2004 and 16 % in 2005–10 [15]. A study in Finland among pregnant women observed a decrease in HSV-2 seroprevalence from 18 % in 1992 to 11 % in 2012, but this decrease was not statistically significant [28].

HSV-1 seropositivity was only slightly associated with sexual risk behavior in our study. There are several explanations for this. First, perhaps most of the HSV-1 seropositive adults of the general population were infected orolabially instead of genitally. We did repeat the analyses with native Dutch adults only to increase the proportion of HSV-1 acquired during adulthood, but this did not change the results (Additional file 4). Second, some sexual risk determinants concerned the past 6 or 12 months, while seroconversion could have occurred a longer time ago. Sexual risk behavior was also only slightly associated with HSV-2 positivity in our study. This might explain why others found that condom use lowered the risk of HSV-2 acquisition [29] and we did not. Another explanation is that HSV-1 seropositivity is related to sexual risk behaviors that were not asked in the Pienter studies, like oral sex [25]. Because information about specific sexual practices was not available, we could not investigate this.

Comparable with other research, we observed that HSV-1 seropositivity was associated with younger age at sexual debut and that participants who ever had sexual intercourse were more likely to be HSV seropositive, indicating sexual transmission [25, 30, 31].

In addition to the limited association between sexual risk behavior and HSV-1 seropositivity, only 0.8 % of the HSV-1 seropositive and HSV-2 seronegative sexually experienced adults reported a history of genital herpes. This percentage increased from 0.5 % in Pienter-1 to 1.3 % in Pienter-2. In comparison, the proportion of HSV-2 seropositive adults who reported a history of genital herpes was higher (3.8 %), but not as high as reported in literature, for example 14 % in the US and 6 % in Canada [32, 33], perhaps due to missing data. Among all adults who ever had sex 3 % did not respond to the STI history question while among HSV-2 seropositive adults this was 6 %. If people with a history of genital herpes were less likely to respond, we underestimated genital herpes.

Other than sexual risk behavior, HSV-1 seropositivity was associated with female gender, being older, non-Dutch ethnicity and low education level, which is comparable to other settings [24, 25, 31]. Children attending day care were more likely to be HSV-1 seropositive than children not attending day care. Interestingly, while the proportion of children attending day care increased over time, the HSV-1 seroprevalence decreased.

Females, older participants and participants with a Surinamese, Aruban, Antillean or other non-Western ethnicity were more often HSV-2 seropositive, which is comparable with the literature [20, 32]. In contrast to the majority of the literature, in our study, low or moderately educated participants were less likely to be HSV-2 seropositive [24, 32]. A few other studies also reported that low educated people or people living in low socio-economic status areas were less often HSV-2 seropositive [34, 35]. The exact mechanism for this is unclear.

The reduced HSV-1 seroprevalence has several implications. First, since a previous HSV-1 infection decreases the likelihood of symptomatic HSV-2 infections, the reduced HSV-1 seroprevalence will lead to more symptomatic HSV-2 infections, assuming it will have no or little influence on the rate of HSV-2 infections [36]. Second, a higher HSV-1 susceptible population leads to more genital acquisition of HSV-1, which in the third trimester of pregnancy leads to a higher risk of neonatal herpes [1]. Although still low, the incidence of neonatal herpes in the Netherlands has increased [3]. More attention can be paid to the HSV serostatus (including HSV-1) of pregnant women and their partners. For serodiscordant couples the risk of acquiring HSV during pregnancy and possible preventive measures, like antiviral treatment of the infected partner and abstinence of unprotected (oral) sex, can be discussed. Vaccines to prevent HSV are being developed, however to date, vaccine research has focused primarily on HSV-2 and has not been very successful in preventing transmission thus far [37]. To prevent genital and neonatal herpes, vaccines must target both HSV-1 and HSV-2.

## Conclusions

Since HSV-1 seroprevalence has decreased, more adults are susceptible to genital HSV-1, including women of reproductive age. It is important to keep monitoring the development of HSV-1 and HSV-2 infections and associated diseases in the population, specifically for the risk of neonatal herpes, vaccine program management and adequate communication to professionals and public. This can be achieved by measuring HSV in the upcoming Pienter-3 seroepidemiological study as well.

## Abbreviations

aOR, adjusted odds ratio; CI, confidence interval; HIV, human immunodeficiency virus; HSV, herpes simplex virus; OR, odds ratio; STI, sexually transmitted infection

## Additional files

Additional file 1: Demographic, social contact determinants and sexual risk determinants for HSV-1 and HSV-2 seropositivity among children, adults, and adults who ever had sexual intercourse, stratified by Pienter study. (DOC 141 kb) Additional file 2: Joint distribution of HSV-1 and HSV-2 weighted seroprevalence by age and Pienter study. (TIF 6106 kb) Additional file 3: Demographic, social contact determinants and sexual risk determinants for HSV-1 and HSV-2 seropositivity among children, adults, and adults who ever had sexual intercourse, with equivocal samples classified as positive. (DOC 144 kb) Additional file 4: Sexual risk determinants for HSV-1 seropositivity among native Dutch adults who ever had sexual intercourse. (DOC 51 kb) Additional file 5: The effect of converting the number of partners in the past year to the number of partners in the past 6 months. (DOC 50 kb)
