# Supplementary material for: Herpes simplex virus type 1 and type 2 in the Netherlands: seroprevalence, risk factors and changes during a 12-year period
Source: BMC Infect Dis. 2016 Aug 2;16:364. doi: 10.1186/s12879-016-1707-8 (PMC4971663; doi:10.1186/s12879-016-1707-8)
Supplement: Additional file 1: — Demographic, social contact determinants and sexual risk determinants for HSV-1 and HSV-2 seropositivity among children, adults, and adults who ever had sexual intercourse, stratified by Pienter study. (DOC 141 kb) [file 12879_2016_1707_MOESM1_ESM.doc]

**Additional file 1**

Logistic regression analyses to investigate demographic and social contact determinants associated with HSV-1 and HSV-2 seropositivity among children and adults*, stratified by Pienter study

|  | HSV-1 | | | | | | HSV-2 | | |
| --- | --- | --- | --- | --- | --- | --- | --- | --- | --- |
|  | Children* | | | Adults* | | | Adults* | | |
|  | Pienter-1  OR [95% CI]† | Pienter-2  OR [95% CI]† | p | Pienter-1  OR [95% CI]† | Pienter-2  OR [95% CI]† | p | Pienter-1  OR [95% CI]† | Pienter-2  OR [95% CI]† | p |
| **Gender** |  |  |  |  |  |  |  |  |  |
| Men | Ref. | Ref. |  | Ref. | Ref. |  | Ref. | Ref. |  |
| Women | 1.15 [0.90-1.46] | **1.45 [1.20-1.75]** |  | 1.13 [0.93-1.38] | **1.37 [1.15-1.63]** |  | **1.36 [1.07-1.74]** | **1.61 [1.21-2.12]** |  |
| **Age (continue)** | **1.21 [1.16-1.25]** | **1.21 [1.18-1.25]** |  | **1.07 [1.05-1.08]** | **1.05 [1.04-1.06]** |  | **1.04 [1.03-1.05]** | **1.07 [1.04-1.09]** |  |
| **Ethnicity** |  |  |  |  |  |  |  |  |  |
| Native Dutch | Ref. | Ref. |  | Ref. | Ref. |  | Ref. | Ref. |  |
| Western, other | 1.27 [0.78-2.07] | 0.54 [0.19-1.54] |  | **1.57 [1.10-2.25]** | 1.24 [0.90-1.71] |  | 1.34 [0.86-2.10] | 1.14 [0.65-1.99] |  |
| Moroccan/  Turkish | **2.48 [1.30-4.72]** | **6.13 [4.16-9.03]** | ‡ | **50.60 [10.27-249.40]** | **9.85 [4.49-21.61]** |  | 0.77 [0.24-2.54] | 0.77 [0.27-2.15] |  |
| Surinamese/ Aruban/Antillean | 1.82 [0.81-4.06] | **3.20 [2.34-4.38]** |  | **3.39 [1.46-7.88]** | **2.21 [1.21-4.02]** |  | **2.85 [1.23-6.59]** | 1.29 [0.57-2.93] |  |
| Non-Western, other | 1.68 [0.86-3.29] | **3.63 [2.42-5.45]** |  | **4.97 [1.56-15.80]** | **4.59 [2.45-8.62]** |  | 2.37 [0.82-6.88] | 1.74 [0.91-3.33] |  |
| **Generation of migrant**** | |  |  |  |  |  |  |  |  |
| Native Dutch | Ref. | Ref. |  | Ref. | Ref. |  | Ref. | Ref. |  |
| 1^st^ generation | **2.47 [1.35-4.50]** | **4.69 [3.27-6.73]** |  | **8.16 [4.77-13.97]** | **3.94 [2.67-5.82]** | ‡ | 1.58 [0.90-2.78] | **1.58 [1.00-2.49]** |  |
| 2^nd^ generation | **1.55 [1.02-2.36]** | **2.64 [1.83-3.80]** |  | 1.38 [0.96-1.98] | 1.43 [0.94-2.17] |  | 1.40 [0.80-2.43] | 0.81 [0.43-1.49] |  |
| **Degree of urbanization** | |  |  |  |  |  |  |  |  |
| Very high | **1.55 [1.03-2.35]** | 0.94 [0.72-1.23] |  | 0.99 [0.71-1.39] | 0.89 [0.74-1.07] |  | 1.22 [0.79-1.90] | 0.96 [0.58-1.60] |  |
| Less high | Ref. | Ref. |  | Ref. | Ref. |  | Ref. | Ref. |  |
| **Education level**†† |  |  |  |  |  |  |  |  |  |
| Moderate/low | **1.63 [1.23-2.16]** | 1.19 [0.85-1.65] |  | 1.21 [0.95-1.54] | 1.22 [0.97-1.54] |  | **0.53 [0.38-0.73]** | 0.88 [0.64-1.23] | ‡ |
| High | Ref. | Ref. |  | Ref. | Ref. |  | Ref. | Ref. |  |
| Unknown | 1.75 [0.63-4.87] | **2.17 [1.17-4.04]** |  | 0.93 [0.38-2.29] | 1.71 [0.65-4.53] |  | 0.52 [0.13-2.10] | 2.16 [0.56-8.26] |  |
| **Household** |  |  |  |  |  |  |  |  |  |
| 1-2 persons | Ref. | Ref. |  | Ref. | Ref. |  | - | - |  |
| 3-4 persons | 0.76 [0.32-1.81] | **2.35 [1.10-5.02]** |  | 1.14 [0.96-1.36] | 1.21 [0.97-1.51] |  | - | - |  |
| >=5 persons | 0.87 [0.34-2.22] | 2.10 [0.97-4.58] |  | 0.92 [0.70-1.20] | 1.27 [0.97-1.66] |  | - | - |  |
| Unknown | 1.31 [0.38-4.51] | **6.02 [3.04-11.93]** | ‡ | 0.89 [0.41-1.94] | 1.64 [0.61-4.37] |  | - | - |  |
| **Child in household attending daycare** | | |  |  |  |  |  |  |  |
| No | Ref. | Ref. |  | Ref. | Ref. |  | - | - |  |
| Yes | 0.76 [0.51-1.13] | 1.20 [0.83-1.73] |  | 1.06 [0.82-1.38] | **1.39 [1.08-1.79]** |  | - | - |  |
| Unknown | 0.68 [0.27-1.71] | **1.94 [1.32-2.87]** | ‡ | **0.35 [0.18-0.72]** | 0.65 [0.30-1.40] |  | - | - |  |
| **Child attending daycare** | |  |  |  |  |  |  |  |  |
| No | Ref. | Ref. |  | - | - |  | - | - |  |
| Yes | **1.46 [1.04-2.05]** | 1.45 [0.93-2.23] |  | - | - |  | - | - |  |
| Unknown | 1.31 [0.60-2.85] | 1.13 [0.73-1.76] |  | - | - |  | - | - |  |
| **Ever had sexual intercourse** | |  |  |  |  |  |  |  |  |
| No | - | - |  | Ref. | Ref. |  | Ref. | Ref. |  |
| Yes | - | - |  | **1.41 [1.02-1.96]** | **1.85 [1.32-2.60]** |  | 2.17 [0.88-5.38] | **2.77 [1.02-7.53]** |  |
| Unknown | - | - |  | 0.89 [0.55-1.44] | **2.17 [1.33-3.54]** | ‡ | 1.44 [0.48-4.33] | **4.03 [1.43-11.39]** |  |
| * Children were aged 6 months to 11 years and adults were aged 17 to 44 years in Pienter-1 and 15 to 44 years in Pienter-2  † OR adjusted for: gender, age, ethnicity and degree of urbanization  ‡ There was a statistically significant (p<0.05) difference between Pienter-1 and Pienter-2  ** Not adjusted for ethnicity  †† For children, the education level of the parents was used  Logistic regression analyses were unweighted, corrected for the complex survey design  In bold: OR is statistically significant (p<0.05)  HSV: Herpes Simplex Virus; OR: Odds Ratio; aOR: adjusted Odds Ratio; CI: confidence interval; Ref: reference | | | | | | | | | |

Logistic regression analyses to investigate sexual risk behavior determinants associated with HSV-1 and HSV-2 seropositivity among adults* who ever had sexual intercourse, stratified by Pienter study

|  | HSV-1 | | |  | HSV-2 | | |
| --- | --- | --- | --- | --- | --- | --- | --- |
|  | Pienter-1  OR [95% CI]† | Pienter-2  OR [95% CI]† | p |  | Pienter-1  OR [95% CI]† | Pineter-2  OR [95% CI]† | p |
| **Number of recent partners**** |  |  |  |  |  |  |  |
| 0 partners | Ref. | Ref. |  |  | Ref. | Ref. |  |
| 1 partners | 1.19 [0.76-1.87] | **1.45 [1.01-2.06]** |  |  | 1.23 [0.64-2.36] | 1.18 [0.62-2.23] |  |
| >=2 partners | 0.88 [0.48-1.62] | 1.32 [0.74-2.38] |  |  | 0.86 [0.30-2.45] | **2.16 [1.04-4.50]** |  |
| Unknown | 1.06 [0.53-2.10] | **2.47 [1.43-4.26]** |  |  | 1.69 [0.70-4.04] | 0.65 [0.22-1.92] |  |
| **Sexual preference** |  |  |  |  |  |  |  |
| Heterosexual | Ref. | Ref. |  |  | Ref. | Ref. |  |
| Homo-/bisexual | 1.19 [0.67-2.12] | 1.05 [0.46-2.39] |  |  | **3.75 [1.69-8.36]** | 1.19 [0.43-3.26] |  |
| Unknown | 0.78 [0.49-1.25] | 0.83 [0.64-1.07] |  |  | 0.75 [0.40-1.39] | **0.52 [0.28-0.97]** |  |
| **Self-reported history of STI** |  |  |  |  |  |  |  |
| No | Ref. | Ref. |  |  | Ref. | Ref. |  |
| Yes, excluding genital herpes | 1.22 [0.66-2.23] | 1.17 [0.69-1.98] |  |  | 1.91 [0.72-5.04] | **1.72 [1.09-2.69]** |  |
| Yes, genital herpes | 1.46 [0.32-6.67] | 2.23 [0.75-6.65] |  |  | **10.14 [2.08-49.49]** | **6.60 [2.49-17.50]** |  |
| Unknown | 1.20 [0.73-1.97] | 0.86 [0.37-1.99] |  |  | 0.87 [0.38-2.00] | 1.73 [0.58-5.18] |  |
| **Age at sexual debut** |  |  |  |  |  |  |  |
| <=16 years | Ref. | Ref. |  |  | Ref. | Ref. |  |
| 17-20 years | 0.85 [0.69-1.07] | **0.64 [0.50-0.81]** | ‡ |  | 1.06 [0.69-1.61] | 1.36 [0.84-2.21] |  |
| >=21 years | **0.65 [0.49-0.85]** | **0.60 [0.46-0.78]** |  |  | 1.06 [0.67-1.67] | 0.99 [0.62-1.56] |  |
| Unknown | 0.93 [0.71-1.23] | 0.82 [0.60-1.11] |  |  | 0.94 [0.56-1.58] | 0.80 [0.40-1.59] |  |
| * Adults were aged 17 to 44 years in Pienter-1 and 15 to 44 years in Pienter-2  † OR adjusted for: gender, age, ethnicity and degree of urbanization  ‡ There was a statistically significant (p<0.05) difference between Pienter-1 and Pienter-2  ** Number of partners in the past year for Pienter-1 and in the past 6 months for Pienter-2  Logistic regression analyses were unweighted, corrected for the complex survey design  In bold: OR is statistically significant (p<0.05)  HSV: Herpes Simplex Virus; OR: Odds Ratio; aOR: adjusted Odds Ratio; CI: confidence interval; Ref: reference; STI: sexually transmitted infection | | | | | | |  |
