# Supplementary material for: Herpes simplex virus type 1 and type 2 in the Netherlands: seroprevalence, risk factors and changes during a 12-year period
Source: BMC Infect Dis. 2016 Aug 2;16:364. doi: 10.1186/s12879-016-1707-8 (PMC4971663; doi:10.1186/s12879-016-1707-8)
Supplement: Additional file 3: — Demographic, social contact determinants and sexual risk determinants for HSV-1 and HSV-2 seropositivity among children, adults, and adults who ever had sexual intercourse, with equivocal samples classified as positive. (DOC 144 kb) [file 12879_2016_1707_MOESM3_ESM.doc]

**Additional file 3**

Sensitivity analyses: logistic regression analyses to investigate demographic and social contact determinants associated with HSV-1 and HSV-2 seropositivity among children and adults*, with equivocal samples classified as positive

|  | HSV-1 | | | | HSV-2 | |
| --- | --- | --- | --- | --- | --- | --- |
|  | Children* | | Adults* | | Adults* | |
|  | OR [95% CI]† | aOR [95% CI] | OR [95% CI]† | aOR [95% CI] | OR [95% CI]† | aOR [95% CI] |
| **Pienter** |  |  |  |  |  |  |
| Pienter-1 | Ref. | Ref. | Ref. | Ref. | Ref. | Ref. |
| Pienter-2 | **0.59 [0.48-0.73]** | **0.59 [0.47-0.73]** | **0.69 [0.58-0.83]** | **0.71 [0.59-0.85]** | 0.85 [0.64-1.11] | 0.82 [0.62-1.08] |
| **Gender** |  |  |  |  |  |  |
| Men | Ref. | Ref. | Ref. | Ref. | Ref. | Ref. |
| Women | **1.27 [1.08-1.50]** | **1.31 [1.11-1.56]** | **1.25 [1.10-1.41]** | **1.22 [1.07-1.39]** | **1.42 [1.17-1.71]** | **1.42 [1.17-1.72]** |
| **Age (continue)** | **1.20 [1.18-1.23]** | **1.23 [1.20-1.26]** | **1.06 [1.05-1.07]** | **1.05 [1.04-1.06]** | **1.05 [1.03-1.06]** | **1.03 [1.02-1.05]** |
| **Ethnicity** |  |  |  |  |  |  |
| Native Dutch | Ref. | Ref. | Ref. | Ref. | Ref. | Ref. |
| Western, other | 0.96 [0.61-1.51] | 0.97 [0.62-1.52] | **1.28 [1.01-1.60]** | **1.36 [1.08-1.72]** | 1.11 [0.77-1.61] | 1.11 [0.77-1.61] |
| Moroccan/  Turkish | **3.63 [2.51-5.25]** | **3.84 [2.62-5.63]** | **16.86 [8.37-33.96]** | **17.59 [8.44-36.68]** | 0.71 [0.32-1.57] | 0.77 [0.33-1.75] |
| Surinamese/ Aruban/Antillean | **2.08 [1.53-2.82]** | **2.52 [1.84-3.46]** | **2.24 [1.38-3.63]** | **2.54 [1.57-4.12]** | 1.70 [0.94-3.07] | **1.88 [1.05-3.39]** |
| Non-Western, other | **2.52 [1.89-3.37]** | **2.82 [2.04-3.91]** | **4.54 [2.70-7.63]** | **5.07 [2.91-8.83]** | 1.69 [0.95-2.98] | **1.83 [1.04-3.23]** |
| **Generation of migrant**‡ | |  |  |  |  |  |
| Native Dutch | Ref. | - | Ref. | - | Ref. | - |
| 1^st^ generation | **3.06 [2.21-4.23]** | - | **4.38 [3.16-6.07]** | - | **1.46 [1.02-2.08]** | - |
| 2^nd^ generation | **1.87 [1.42-2.45]** | - | **1.37 [1.05-1.79]** | - | 0.94 [0.61-1.45] | - |
| **Degree of urbanization** | |  |  |  |  |  |
| Very high | 1.11 [0.82-1.49] | 1.16 [0.90-1.50] | 0.88 [0.70-1.10] | 0.97 [0.81-1.17] | 1.00 [0.69-1.43] | 0.99 [0.69-1.42] |
| Less high | Ref. | Ref. | Ref. | Ref. | Ref. | Ref. |
| **Education level**** |  |  |  |  |  |  |
| Moderate or low | **1.41 [1.13-1.76]** | **1.41 [1.13-1.77]** | **1.34 [1.14-1.56]** | **1.28 [1.09-1.51]** | **0.73 [0.58-0.92]** | **0.72 [0.57-0.92]** |
| High | Ref. | Ref. | Ref. | Ref. | Ref. | Ref. |
| Unknown | **2.22 [1.26-3.90]** | 1.64 [0.86-3.14] | 1.27 [0.63-2.56] | 1.67 [0.78-3.59] | 1.05 [0.41-2.70] | 1.08 [0.40-2.93] |
| **Household** |  |  |  |  |  |  |
| 1-2 persons | Ref. | Ref. | Ref. | Ref. | - | - |
| 3-4 persons | 1.72 [0.93-3.17] | 1.70 [0.92-3.14] | **1.16 [1.02-1.32]** | **1.15 [1.01-1.30]** | - | - |
| >=5 persons | 1.70 [0.90-3.21] | 1.74 [0.93-3.28] | 1.08 [0.90-1.29] | 1.11 [0.91-1.35] | - | - |
| Unknown | **4.33 [2.17-8.61]** | **4.18 [1.91-9.17]** | 1.11 [0.62-1.99] | **2.06 [1.02-4.18]** | - | - |
| **Child in household attending daycare** | | |  |  |  |  |
| No | Ref. | Ref. | Ref. | Ref. | - | - |
| Yes | 0.89 [0.66-1.20] | 0.90 [0.68-1.19] | 1.09 [0.90-1.32] | 1.07 [0.87-1.31] | - | - |
| Unknown | **1.49 [1.01-2.19]** | 1.33 [0.64-2.75] | **0.46 [0.28-0.76]** | **0.32 [0.17-0.57]** | - | - |
| **Child attending daycare** | |  |  |  |  |  |
| No | Ref. | Ref. | - | - | - | - |
| Yes | **1.43 [1.11-1.86]** | **1.55 [1.17-2.05]** | - | - | - | - |
| Unknown | 1.30 [0.87-1.95] | 0.71 [0.33-1.55] | - | - | - | - |
| **Ever had sexual intercourse** | |  |  |  |  |  |
| No | - | - | Ref. | Ref. | Ref. | Ref. |
| Yes | - | - | **1.67 [1.33-2.09]** | **1.74 [1.38-2.20]** | **2.44 [1.32-4.51]** | **2.33 [1.27-4.30]** |
| Unknown | - | - | **1.43 [1.03-1.98]** | **1.45 [1.03-2.04]** | **2.34 [1.13-4.84]** | **2.15 [1.03-4.48]** |
| * Children were aged 6 months to 11 years and adults were aged 17 to 44 years in Pienter-1 and 15 to 44 years in Pienter-2  † OR adjusted for: gender, age, ethnicity and degree of urbanization  ‡ Not adjusted for ethnicity and not included in multivariable analyses  ** For children, the education level of the parents was used  Logistic regression analyses were unweighted, corrected for the complex survey design  In bold: OR is statistically significant (p<0.05)  HSV: Herpes Simplex Virus; OR: Odds Ratio; aOR: adjusted Odds Ratio; CI: confidence interval; Ref: reference | | | | | | |

Sensitivity analyses: logistic regression analyses to investigate sexual risk determinants associated with HSV-1 and HSV-2 seropositivity among adults* who ever had sexual intercourse, with equivocal samples classified as positive

|  | HSV-1 | | HSV-2 | |
| --- | --- | --- | --- | --- |
|  | OR [95% CI]† | aOR [95% CI]‡ | OR [95% CI]† | aOR [95% CI]‡ |
| **Number of recent partners**** |  |  |  |  |
| 0 partners | Ref. | Ref. | Ref. | Ref. |
| 1 partners | **1.34 [1.01-1.78**] | 1.13 [0.72-1.78] | 1.30 [0.84-2.02] | 0.51 [0.25-1.05] |
| >=2 partners | 1.16 [0.76-1.75] | 0.89 [0.52-1.51] | 1.55 [0.87-2.73] | 0.50 [0.22-1.10] |
| Unknown | **1.82 [1.20-2.78]** | 1.48 [0.94-2.33] | 1.41 [0.76-2.62] | 0.85 [0.41-1.75] |
| **Sexual preference** |  |  |  |  |
| Heterosexual | Ref. | Ref. | Ref. | Ref. |
| Homo-/bisexual | 1.01 [0.61-1.67] | 1.10 [0.65-1.88] | **2.13 [1.14-3.99]** | 1.78 [0.93-3.42] |
| Unknown | 0.81 [0.64-1.03] | 0.93 [0.64-1.36] | **0.58 [0.38-0.87]** | **0.35 [0.18-0.67]** |
| **Self-reported history of STI** |  |  |  |  |
| No | Ref. | Ref. | Ref. | Ref. |
| Yes, excluding genital herpes | 1.16 [0.77-1.76] | 1.18 [0.78-1.78] | **1.65 [1.00-2.71]** | 1.57 [0.93-2.65] |
| Yes, genital herpes | **2.55 [1.06-6.11]** | **2.58 [1.06-6.27]** | **6.98 [3.00-16.25]** | **7.21 [3.04-17.10]** |
| Unknown | 1.18 [0.78-1.77] | 1.07 [0.72-1.59] | 1.03 [0.56-1.92] | 1.09 [0.60-1.98] |
| **Age at sexual debut** |  |  |  |  |
| <=16 years | Ref. | Ref. | Ref. | Ref. |
| 17-20 years | **0.76 [0.64-0.90]** | **0.75 [0.63-0.89]** | 1.21 [0.89-1.63] | 1.22 [0.89-1.68] |
| >=21 years | **0.63 [0.50-0.78]** | **0.64 [0.51-0.81]** | 1.02 [0.74-1.40] | 1.03 [0.73-1.45] |
| Unknown | 0.89 [0.72-1.11] | 0.83 [0.68-1.03] | 0.90 [0.61-1.33] | 0.92 [0.61-1.37] |
| **Condom use steady partner**†† |  |  |  |  |
| Consistent | Ref. | - | Ref. | - |
| Inconsistent | 0.94 [0.69-1.29] | - | 1.08 [0.65-1.78] | - |
| Unknown/no steady partner | 0.91 [0.65-1.28] | - | 0.78 [0.44-1.39] | - |
| **Condom use casual partner**†† |  |  |  |  |
| Consistent | Ref. | - | Ref. | - |
| Inconsistent | 1.26 [0.68-2.34] | - | 0.30 [0.07-1.29] | - |
| Unknown/no casual partner | 1.07 [0.68-1.69] | - | 0.65 [0.33-1.31] | - |
| * Adults were aged 17 to 44 years in Pienter-1 and 15 to 44 years in Pienter-2  † OR adjusted for: gender, age, ethnicity and degree of urbanization  ‡ Adjusted for all variables including those presented in Table 2  ** Number of partners in the past year for Pienter-1 and in the past 6 months for Pienter-2  †† Condom use in the past 6 months. Available for Pienter-2 only  Logistic regression analyses were unweighted, corrected for the complex survey design  In bold: OR is statistically significant (p<0.05)  HSV: Herpes Simplex Virus; OR: Odds Ratio; aOR: adjusted Odds Ratio; CI: confidence interval; Ref: reference; STI: sexually transmitted infection | | | | |
