# Supplementary material for: Herpes simplex virus type 1 and type 2 in the Netherlands: seroprevalence, risk factors and changes during a 12-year period
Source: BMC Infect Dis. 2016 Aug 2;16:364. doi: 10.1186/s12879-016-1707-8 (PMC4971663; doi:10.1186/s12879-016-1707-8)
Supplement: Additional file 4: — Sexual risk determinants for HSV-1 seropositivity among native Dutch adults who ever had sexual intercourse. (DOC 51 kb) [file 12879_2016_1707_MOESM4_ESM.doc]

**Additional file 4**

Sensitivity analyses: Logistic regression analyses to investigate sexual risk determinants associated with HSV-1 seropositivity among native Dutch adults* who ever had sexual intercourse

|  | HSV-1 | |
| --- | --- | --- |
|  | OR [95% CI]† | aOR [95% CI]‡ |
| **Number of recent partners**** |  |  |
| 0 partners | Ref. | Ref. |
| 1 partners | **1.37 [1.02-1.86]** | 1.11 [0.68-1.81] |
| >=2 partners | 1.12 [0.74-1.71] | 0.82 [0.47-1.44] |
| Unknown | **1.63 [1.02-2.61]** | 1.31 [0.76-2.23] |
| **Sexual preference** |  |  |
| Heterosexual | Ref. | Ref. |
| Homo-/bisexual | 1.10 [0.64-1.87] | 1.27 [0.73-2.22] |
| Unknown | **0.77 [0.60-0.99]** | 0.87 [0.58-1.32] |
| **Self-reported history of STI** |  |  |
| No | Ref. | Ref. |
| Yes, excluding genital herpes | 1.05 [0.67-1.64] | 1.06 [0.67-1.67] |
| Yes, genital herpes | 2.05 [0.72-5.86] | 2.04 [0.69-6.04] |
| Unknown | 1.05 [0.67-1.65] | 1.00 [0.63-1.58] |
| **Age at sexual debut** |  |  |
| <=16 years | Ref. | Ref. |
| 17-20 years | **0.73 [0.61-0.88]** | **0.72 [0.60-0.87]** |
| >=21 years | **0.63 [0.51-0.78]** | **0.64 [0.51-0.81]** |
| Unknown | 0.84 [0.69-1.04] | 0.82 [0.66-1.01] |
| **Condom use steady partner**†† |  |  |
| Consistent | Ref. | - |
| Inconsistent | 0.95 [0.69-1.29] | - |
| Unknown/no steady partner | 0.87 [0.57-1.32] | - |
| **Condom use casual partner**†† |  |  |
| Consistent | Ref. | - |
| Inconsistent | 1.55 [0.79-3.03] | - |
| Unknown/no casual partner | 1.08 [0.69-1.72] | - |
| * Adults were aged 17 to 44 years in Pienter-1 and 15 to 44 years in Pienter-2. Adults with at least one parent born abroad were excluded  † OR adjusted for: gender, age, ethnicity and degree of urbanization  ‡ Adjusted for all variables including those presented in Table 2  ** Number of partners in the past year for Pienter-1 and in the past 6 months for Pienter-2  †† Condom use in the past 6 months. Available for Pienter-2 only  Number and percentages were unweighted. Logistic regression analyses were unweighted, corrected for the complex survey design  In bold: OR is statistically significant (p<0.05)  HSV: Herpes Simplex Virus; OR: Odds Ratio; aOR: adjusted Odds Ratio; CI: confidence interval; Ref: reference; STI: sexually transmitted infection | | |
