# Supplementary material for: Herpes simplex virus type 1 and type 2 in the Netherlands: seroprevalence, risk factors and changes during a 12-year period
Source: BMC Infect Dis. 2016 Aug 2;16:364. doi: 10.1186/s12879-016-1707-8 (PMC4971663; doi:10.1186/s12879-016-1707-8)
Supplement: Additional file 5: — The effect of converting the number of partners in the past year to the number of partners in the past 6 months. (DOC 50 kb) [file 12879_2016_1707_MOESM5_ESM.doc]

**Additional file 5**

Sensitivity analyses to investigate the effect of converting the number of partners in the past year (Pienter-1 variable) to the number of partners in the past 6 months (Pienter-2 variable) on HSV-1 and HSV-2 seropositivity

|  | HSV-1 | | | | |
| --- | --- | --- | --- | --- | --- |
|  | Pienter-1 | Pienter-2 |  | Pienter-1 and 2 | Pienter-1 and 2 |
|  | OR [95% CI]* | OR [95% CI]* | p | OR [95% CI]* | aOR [95% CI]† |
| **Number of partners in the past 6 months**** |  |  |  |  |  |
| 0 partners | Ref. | Ref. |  | Ref. | Ref. |
| 1 partners | 1.17 [0.74-1.84] | **1.45 [1.02-2.07]** |  | **1.38 [1.04-1.82]** | 1.10 [0.71-1.73] |
| >=2 partners | 1.12 [0.54-2.31] | 1.33 [0.74-2.39] |  | 1.24 [0.78-1.96] | 0.95 [0.53-1.71] |
| Unknown | 1.07 [0.54-2.12] | **2.49 [1.44-4.29]** | ‡ | **1.69 [1.10-2.60]** | 1.34 [0.84-2.14] |
|  |  |  |  |  |  |
|  | HSV-2 | | | | |
|  | Pienter-1 | Pienter-2 |  | Pienter-1 and 2 | Pienter-1 and 2 |
|  | OR [95% CI]* | OR [95% CI]* | p | OR [95% CI]* | aOR [95% CI]† |
| **Number of partners in the past 6 months**** |  |  |  |  |  |
| 0 partners | Ref. | Ref. |  | Ref. | Ref. |
| 1 partners | 1.21 [0.63-2.31] | 1.17 [0.62-2.21] |  | 1.27 [0.80-2.01] | 0.51 [0.24-1.09] |
| >=2 partners | 1.41 [0.32-6.28] | **2.15 [1.03-4.48]** |  | **1.96 [1.01-3.82]** | 0.61 [0.24-1.54] |
| Unknown | 1.71 [0.71-4.10] | 0.64 [0.22-1.91] |  | 1.27 [0.65-2.47] | 0.77 [0.37-1.62] |
| * OR adjusted for: gender, age, ethnicity and degree of urbanization.  † Adjusted for all variables presented in Tables 2 and 3.  ‡ There was a statistically significant (p<0.05) difference between Pienter-1 and Pienter-2.  ** The number of sex partners in the past year (Pienter-1 variable) was converted to the number of partners in the past 6 months, by rounding up the number of partners in the past year divided by 2.  Only adults who ever had sexual intercourse were included. Adults were aged 17 to 44 years in Pienter-1 and 15 to 44 years in Pienter-2.  Logistic regression analyses were unweighted, corrected for the complex survey design.  In bold: OR is statistically significant (p<0.05).  HSV: Herpes Simplex Virus; OR: Odds Ratio; aOR: adjusted Odds Ratio; CI: confidence interval; Ref: reference; STI: sexually transmitted infection. | | | | | |
